# CNN-BLPred: a Convolutional neural network based predictor for β-Lactamases (BL) and their classes

**DOI:** 10.1186/s12859-017-1972-6

**Published:** 2017-12-28

**Authors:** Clarence White, Hamid D. Ismail, Hiroto Saigo, Dukka B. KC

**Affiliations:** 1 Department of Computational Science and Engineering, North Carolina A&T State University, Greensboro, NC 27411 USA; 2 Faculty of Information Science and Electrical Engineering, Kyushu University, 744 Motooka, Nishi-ku, Fukuoka, 819-0395 Japan

**Keywords:** Beta lactamase protein classification, Feature selection, Convolutional neural network, Deep learning

## Abstract

**Background:**

The β-Lactamase (BL) enzyme family is an important class of enzymes that plays a key role in bacterial resistance to antibiotics. As the newly identified number of BL enzymes is increasing daily, it is imperative to develop a computational tool to classify the newly identified BL enzymes into one of its classes. There are two types of classification of BL enzymes: Molecular Classification and Functional Classification. Existing computational methods only address Molecular Classification and the performance of these existing methods is unsatisfactory.

**Results:**

We addressed the unsatisfactory performance of the existing methods by implementing a Deep Learning approach called Convolutional Neural Network (CNN). We developed CNN-BLPred, an approach for the classification of BL proteins. The CNN-BLPred uses Gradient Boosted Feature Selection (GBFS) in order to select the ideal feature set for each BL classification. Based on the rigorous benchmarking of CCN-BLPred using both leave-one-out cross-validation and independent test sets, CCN-BLPred performed better than the other existing algorithms.

Compared with other architectures of CNN, Recurrent Neural Network, and Random Forest, the simple CNN architecture with only one convolutional layer performs the best. After feature extraction, we were able to remove ~95% of the 10,912 features using Gradient Boosted Trees. During 10-fold cross validation, we increased the accuracy of the classic BL predictions by 7%. We also increased the accuracy of Class A, Class B, Class C, and Class D performance by an average of 25.64%. The independent test results followed a similar trend.

**Conclusions:**

We implemented a deep learning algorithm known as Convolutional Neural Network (CNN) to develop a classifier for BL classification. Combined with feature selection on an exhaustive feature set and using balancing method such as Random Oversampling (ROS), Random Undersampling (RUS) and Synthetic Minority Oversampling Technique (SMOTE), CNN-BLPred performs significantly better than existing algorithms for BL classification.

**Electronic supplementary material:**

The online version of this article (10.1186/s12859-017-1972-6) contains supplementary material, which is available to authorized users.

## Background

### β*-lactamases family*

β-lactam antibiotics are an important class of drugs that are used to treat various pathogenic bacteria to treat bacterial infections. However, over the course of time, bacteria naturally develop resistance against antibiotics. Antibiotic resistance continues to threaten our ability to cope with the pace of development of new antibiotic drugs [1].

One of the major bacterial enzymes that hinders the effort to produce new antibiotic drugs of the β-lactam family is the β-lactamase (BL) enzyme. The BL enzyme family has a chemically diverse set of substrates. BL develops resistance to penicillin and related antibiotics by hydrolyzing their conserved 4-atom β-lactam moiety, thus destroying their antibiotic activity [2]. β-lactam antibiotics effectively inhibit bacterial transpeptidases, hence, they are also referred to as penicillin binding proteins (PBP). Bacteria have evolved BL enzymes to defend themselves against B-lactam antibiotics. This transformation causes the BL enzyme family to have varying degrees of antibiotic resistance activity. Once a BL enzyme is identified, it can be inhibited by a drug known as clavulanic acid. Clavulanic acid is a naturally produced BL inhibitor discovered in 1976, and when combined with β-lactams, it prevents hydrolysis of the Beta-Lactams. Pathogens develop resistance by modifying or replacing the target proteins and acquiring new BLs. This results in an increasing number of BLs, BL variants, and a widening gap between newly discovered BL protein sequences and their annotations.

**Fig. 1:**
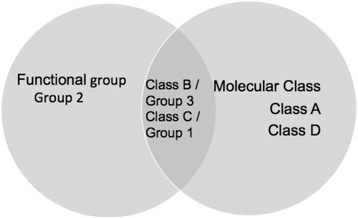
Venn diagram showing the relationship between molecular class and Functional group of Beta Lactamase

The current classification schemes for BL enzymes are molecular classification and functional grouping. The molecular classes are A, B, C, and D. Class A, C, and D act by serine-based mechanism, while Class B requires zinc as a precursor for activation. Bush et al. originally proposed three functional groups in 1995: Group 1, Group 2 and Group 3. More recently [3], the functional grouping scheme has been updated to correlate them with their phenotype in clinical isolates. The updated classification Group 1 (Cephalosporinases) contains molecular Class C which is not inhibited by clavulanic acid and contains a subgroup called 1e. Group 2 (Serine BLs) contains molecular Classes A and D, which are inhibited by clavulanic acid and contain subgroups 2a, 2b, 2be, 2br, 2ber, 2c, 2ce, 2d, 2de, 2df, 2e, and 2 f. Group 3 (Metallo-b-lactamases [MBLs]) contains molecular Class B, which is not inhibited by clavulanic acid and contains subclasses B1, B2, and B3 and subgroups 3a, 3b and 3c. A simple Venn diagram showing the relationship between molecular class and functional groups is shown in Fig. 1.

Numerous studies have been performed to categorize all the classes of BL and their associated variants, along with their epidemiology and resistance pattern information [4–6]. One of these resources is the β-Lactamase Database (BLAD) [5], which contains BL sequences linked with structural data, phenotypic data, and literature references to experimental studies. BLAD contains more than 1154 BL enzymes identified as of July 2015 [7], which are classified into 4 classes [A, B, C and D] based on sequence similarity [8]. Similarly, these proteins have also been divided into classes based on functional characteristics [9]. BL belonging to classes A, C, and D have similar folds and a mechanism that involves a catalytic serine residue whereas class B of BL has a distinct fold [7]. It is possible to detect the presence of BL enzymes by conducting various biological experiments; however, it is both time-consuming and costly. Hence, the development of computational methods to predict the identification and classification of BLs is a strong alternative approach to aid in the annotation of BL.

Few computational studies have been conducted in order to predict the BL proteins classes. Srivastava et al. proposed a fingerprint (unique family specific motif) based method to predict the family of BLs [10]. As this method relies on extracting motifs in the sequences, there is inherent limitations when looking specifically for conserved motifs. Subsequently, Kumar et al. proposed a support vector machine based approach for prediction of BL classes [11]. This method uses Chou’s pseudo-amino acid composition [12] and is a two-level BL prediction method. The first level predicts whether or not a given sequence is a BL and if so, the second level classifies the BL into different classes. This method identifies BL with sufficient accuracy, but underperforms in classification accuracy.

### Feature extraction

We recently developed a comprehensive Feature Extraction from Protein Sequences (FEPS) web server [13]. FEPS uses published feature extraction methods of proteins from single or multiple-FASTA formatted files. In addition, FEPS also provides users the ability to redefine some of the features by choosing one of the 544 physicochemical properties or to enter any user-defined amino acid indices, thereby increasing feature choices. The FEPS server includes 48 published feature extraction methods, six of which can use any of the 544 physicochemical properties. The total number of features calculated by FEPS is 2765, which exceeds the number of features computed by any other peer application. This exhaustive list of feature extraction methods enables us to develop machine learning based approaches for various classification problems in bioinformatics. FEPS has been successfully applied for the prediction and classification of nuclear receptors [13], prediction of phosphorylation sites [14], and prediction of hydroxylation sites [15].

### Convolutional neural network (CNN)

To improve identification and classification of BL enzymes, we implemented a Convolutional Neural Network (CNN) based two-level approach called CNN-BLPred. CNN is a specific type of deep neural network that uses a translation-invariant convolution kernel that can be used to extract local contextual features and has proven to be quite successful in various domains [16] including but not limited to computer vision and image classification, spam topic categorization, sentiment analysis, spam detection, and others [17]. The basic structure of CNNs consists of convolution layers, nonlinear layers, and pooling layers. Recently, CNN has been applied to several bioinformatics problems [18].

Moreover, there exist various balancing techniques like Synthetic Minority Oversampling Technique (SMOTE) [19], random oversampling (ROS), and random undersampling (RUS) to balance the dataset when the number of positive and negative examples is not balanced. It has also been observed in several studies that a balanced dataset provides an improvement in the overall performance for classifiers. In the field of bioinformatics, Wei and Dunbrack [19] studied the effect of unbalanced data and found that balanced training data results in the highest balanced performance.

## Methods

### Beta lactamase family classification

Since BL have two types of classification, molecular classes and functional groups, we designed an algorithm to identify both types of classification. To our knowledge, this is the first computational work dealing with the classification of BL into functional groups.

### Benchmark dataset 1: Molecular class/functional group

BL have been classified into four molecular classes: Class A, Class B, Class C, and Class D. BL have also been classified into three functional groups: 1, 2, and 3.

We used one training dataset for cross-validation and two independent datasets for our testing purposes.

For the first benchmark dataset, the positive BL enzyme sequences were obtained from the NCBI website by using ‘Beta-Lactamase’ as a keyword search term to obtain BL enzyme sequences. In total 1,022,470 sequences were retrieved (as of Feb 2017) and sequences that contained keyword ‘partial’ in the sequence header were removed. Then, the sequences were split into molecular classes using keywords ‘Class A, Class B, Class C, and Class D’. This resulted in 11,987, 120,465, 12,350, and 4583 sequences for Class A, Class B, Class C, and Class D respectively (Table 1). This is summarized in Table 1. For the non-BL enzyme sequences, the same sequences used in PredLactamase [11] were used. These sequences were used as a negative set for our general (Level 1) BL classifier.

**Table 1:** Molecular Class/Functional Group Benchmark Dataset

#	Class/Group	# of Sequences Before /After CD-hit
1	Class A	11,987/278
2	Class B/Group 3	120,465/2184
3	Class C/Group 1	12,350/744
4	Class D	4853/62
5	Group 2	16,840/340
6	Non BL	497

Redundant sequences from each class were removed using CD-HIT (40%) [20]. This resulted in 278 Class A, 2184 Class B (Group 3), 744 Class C (Group 1), and 62 Class D sequences. The 340 Group 2 sequences were derived by combining Class A and D sequences. From these sequences, 95% were used for training and the remaining 5% of the dataset was left out for independent testing (Table 2).

**Table 2:** Molecular Class/Functional Group Datasets

#	Class/Group	Training	Independent 1	Independent 2
1	Class A	268	10	4
2	Class B/Group 3	2069	115	6
3	Class C/Group 1	701	43	6
4	Class D	59	3	4
5	Group 2	318	22	8
6	Non BL	478	19	–

### Independent datasets

An independent dataset is required to assess the blind performance of the method. Our experiment incorporated two independent datasets. The number of sequences in the Independent Dataset 1 (Additional file 1) is shown in Table 2 (created with the remaining 5% of the left out dataset) and we used the independent dataset from PredLactamase [11] as our Independent Dataset 2 (Additional file 2). Using Additional file 2: Independent Dataset 2 allows us to compare our method to the previously published PredLactamase method.

**Fig. 2:**
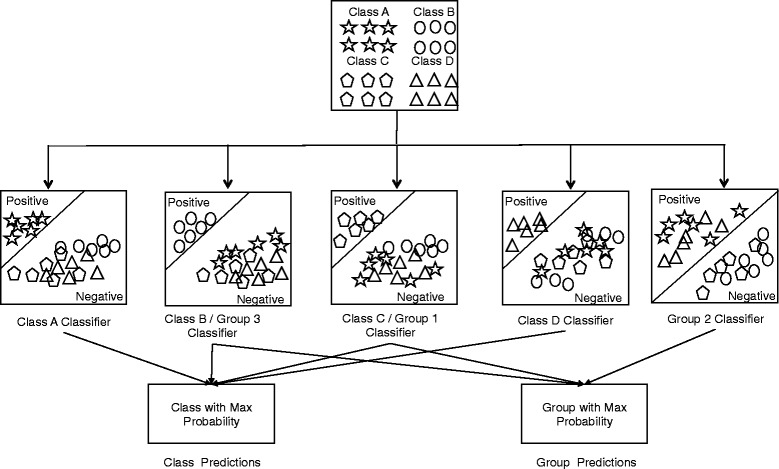
Schematic of our multi-class classification approach for Beta Lactamase

As discussed earlier, our method consists of two steps: identification and classification. The identification step uses the Level 1 predictor and will determine whether a protein is a BL or not. If the protein is not predicted as a BL enzyme during the identification step, the process will stop; otherwise the protein is passed to the next step, which is classification step. During classification, predictors for Classes A and B (aka Group 3), C (aka Group 1), D, and Group 2 are used. This step returns predictions and probabilities for each predictor and we take the prediction with the highest probability for each classification scheme (molecular and functional). Our method returns multiple predictions in the instance of multiple predictors returning the same maximum probabilities. The schematic of the one-vs.-rest classification is depicted in Fig. 2. a set of binary classifiers using a one-vs.-rest strategy, and each resulting molecular class dataset includes data from the other three classes as a negative set. For example, Class A has 278 positive examples and 2990 (total of classes B, C and D) negative examples. Our Group 2 predictor has 318 positive examples and 2770 (total of groups 1 and 2) as negative examples. Our Level 1 predictor has 3268 (total BL sequences) positive examples and 497 negative examples.

### Balanced training data set

Due to the different number of positive and negative training examples (BL enzymes as well as respective BL enzymes belonging to each class), we must resolve class imbalance before moving to classifier training. We balanced our resulting dataset to obtain the optimal accuracy. Some of the techniques that we used to solve this imbalanced dataset problem are random undersampling (RUS), random oversampling (ROS), and Synthetic Minority Oversampling Technique SMOTE [21]. RUS is the procedure of randomly eliminating examples from the majority class until the number of examples matches that of the minority class. RUS does not suffer from the problem of overfitting but can suffer from the loss of potentially useful data. ROS is the opposite of RUS in that it randomly replicates examples of the minority class until it matches that of the majority class. Using ROS, we will not lose potentially useful data; however, the act of randomly replicating data can cause a model to fit too closely to the training data and subsequently overfit. SMOTE is a variation of ROS that solves the overfitting problem by creating synthetic instances instead of making random copies. This method is also useful in that it can extract more information from data that is very helpful when our dataset is small.

For the molecular classes, we utilize ROS for Level 1, Class A, Class C/Group 1 and Group 2 so that we do not discard any potentially useful data. Because we have a significant number of examples of the majority class, we use RUS for Class B/Group 3 to reduce the potential of overfitting. The dataset for Class D is small, so we use SMOTE to maximize the data practicality. The resulting Dataset is shown in Table 3 and is used for training the model.

**Table 3:** Molecular Class/Functional Group Benchmark Dataset after Balancing

#	Class	Method	Positive	Negative
1	Level 1	ROS	3268	3268
2	Class A	ROS	2990	2990
3	Class B/Group 3	RUS	1084	1084
4	Class C/Group 1	ROS	2524	2524
5	Class D	SMOTE	3200	3200
6	Group 2	ROS	2770	2770

### Protein sequence features

Machine learning algorithms, like CNN, work on vectors of numerical values. To classify protein sequences using CNN, we transformed the protein sequences into vectors of numerical values using FEPS. The features we used in our study were: k-Spaced Amino Acid Pairs (CKSAAP), Conjoint Triad (CT), and Tri-peptide Amino Acid Composition (TAAC). CNNs have superior predictive power and are well-equipped to learn “simple” features, however they have limited capabilities for data of mixed types (complex features). Also, feature embedding is typically implemented on continuous vector space with low dimensions. To alleviate these issues, we only evaluate features that contain whole numbers, i.e. CKSAAP, CT, and TAAC. The total number of features considered in the study was 10,912 (Table 4). We describe the features used in this study below.

**Table 4:** Feature set and Feature Selection Results. CSKAAP [22] refers to the K-spaced amino acid Pairs, CT [20] refers to Conjoint Triad and TAAC is the Tri-peptide Amino acid composition

Feature Set	Total Features		Molecular Class / Functional Group – Total Features after Feature Selection				
		Level 1	Class A	Class B / Group 3	Class C / Group 1	Class D	Group 2
CKSAAP [22]	2400	367	270	240	230	197	266
CT [20]	512	208	151	149	145	147	160
TAAC	8000	325	227	262	249	120	219
ALL	10,912	363	288	243	257	195	270

### Tri-peptide amino acid composition (TAAC)

Tri-peptide Amino-Acid Composition (3-mer spectrum) of a sequence represents the frequency of three contiguous amino acids in a protein sequence. In other words, TAAC is the total count of each possible 3-mer of amino acids in the protein sequence. TAAC is defined as below where N is length of the sequence.

1 $$ {f}_j=\frac{\#\mathrm{of}\  \mathrm{tripeptide}\ j}{N-2}\times 100 $$

where *tripeptide*_*j*_ represents any possible tripeptide. The total number of 3-mers is 20^3^ = 8000, *i* = 1,2,3, …8000.

### Conjoint triad

Conjoint triad descriptors (CT) were first described by Shen et al. [22] to predict protein-protein interactions. The conjoint triad descriptors represent the features of protein pairs based on the classification of amino acids. In CTD the properties of one amino acid and its vicinal amino acids and regards any three continuous amino acids as a unit.

To calculate the conjoint triad, originally the amino acids are clustered into seven classes based on their dipole and the volume of the side chain. The newer Conjoint Triad Feature (CTF2) proposed by Yin and Tan [23] includes the dummy amino acid that is used to ensure the identical of the window size of the amino acid sequence. Therefore, the dummy amino acid gets assigned an extra class, which is noted as O. The whole 21 amino acids are thus classified into eight classes: {A, G, V}, {I, L, F, P}, {Y, M, T, S}, {H, N, Q, W}, {R, K}, {D, E}, {C}, {O}. The rest of the encoding method is the same as the CT encoding [22]. The amino acids in the same group are likely to substitute one another because of the physiochemical similarity. One class is added to account for possible ‘dummy’ amino acids that are placed into a sequence. We will refer to this newer Conjoint Triad features as CT in the rest of the paper. For CT, the amino acids are catalogued into eight classes; hence the size of the feature vector for CT is 8x8x8 = 512.

### K-spaced amino-acid pairs (CKSAAP)

k-spaced amino-acid pairs features were originally developed by Chen et al. [24]. Essentially, for a given protein sequence all the adjacent pairs of Amino Acids (AAs) (dipeptides) in the sequence are counted. Since there are 400 possible AA pairs (*AA*, *AC*, *AD*, ..., *YY*), a feature vector of that size is used to represent occurrence of these pairs in the window. In order to accommodate for the short-range interactions between AAs, rather than only interactions between immediately adjacent AAs, CKSAAP also considers k-spaced pairs of AAs, i.e. pairs that are separated by *k* other AAs. For our purpose we use *k* = 0, 1... 5, where for *k* = 0 the pairs reduce to dipeptides. For each value of *k*, there are 400 corresponding features. In total we have 2400 features for CKSAAP. The feature type and number of features in each type is summarized in Table 4. As discussed in the results section, we obtain best results using CKSAAP as the only type of feature. Hence, in CNN-BLPred we represent each protein sequence using CKSAAP only.

### Feature importance and feature selection

Feature importance for our purpose refers to determining the correlation between individual features in our feature set and the class labels. Highly correlated features are very important to our problem and features with low to no correlation are deemed unimportant to our problem. There are generally three method types to determine such importance. The first set of methods is linear methods, such as Lasso. These are easy to implement and scale readily to large dataset. However, as their name implies, linear methods are only able to determine linear correlations between features and provide no insight into non-linear correlations. The next set of methods is kernel methods, such as HSIC Lasso, which are able to determine non-linear correlations. These methods, however, do not scale well to large datasets and will quickly become intractable as the dataset grows. The last method, which is what we have chosen is called tree based methods, such as Gradient Boosted Trees, solves the issues of both previous methods by allowing us to detect non-linear correlations in a scalable way.

Once the features are extracted, we remove the unimportant features from our dataset to improve the overall quality of our model. We use XGBOOST in Python to construct the gradient boosted trees [25]. Since our feature selection method is a tree based method, the feature importance is calculated based on a common metric known as impurity. Impurity is generally used to describe the ability of the feature to cleanly split the input data into the correct class. The equation used in our method is Gini Impurity that is denoted as:

2 $$ G=\sum \limits_{i=1}^{n_c}{p}_i\left(1-{p}_i\right) $$

Where n_c_ is the number class and p_i_ is the probability value of i. Each node in the gradient boosted trees is given a Gini impurity index and this is used to calculate what is called the Gini Importance measure which is calculated as:

3 $$ I={G}_{\mathrm{parent}}-{G}_{\mathrm{split}1}-{G}_{\mathrm{split}2} $$

Any feature with a relative importance value of <0.001 is considered unimportant. Based on this, we were able to classify ~97.5% of the total features (for the combination of all the features) as unimportant and subsequently remove them. Table 4 shows the remaining features after calculating the feature importance and performing feature selection.

### Convolutional neural network (CNN)

**Fig. 3:**
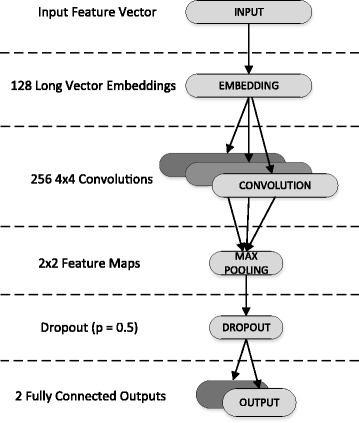
Convolutional Neural Network (CNN) architecture used in our approach

For our CNN, we input a training data set and a corresponding label set (BL or not, class, etc.) and proceeded with the following steps. First, we used the schemes described in earlier section to construct features for each proteins. For each protein, there are 10,912 features. Next, we described the chosen architecture of the CNN for our purpose. The schematic of the architecture is shown in Fig. 3. The first layer of our network is the *input layer*. Our benchmark dataset, which includes the selected features in Table 4, is fed into the input layer of the network which used a stochastic optimization method called Adam (Adaptive Moment Estimation), categorical cross entropy as the loss function, and a learning rate of 0.001.

The next layer of our network is the *embedding layer* [26]. This layer is used to identify semantic similarities between features. Typically, embedding is implemented on a space with one dimension per word or a continuous vector space with low dimensions. The input dimensions of this layer are the length of the feature vector space and the output of 128 embeddings that will be passed into the next layer.

The third layer of our network is the *convolutional layer*, which functions as a motif scanner. CNN-BLPred uses 256 convolutional filters, each scanning the input sequence with a step size of 1 and window size of 4. The output of each neuron on a convolutional layer is the convolution of the kernel matrix and the part of the input within the neuron’s window size. We used tanh activations along with L2-Regularization.

The fourth layer is the *max-pooling layer*. Since convolution output can vary in length, we performed max pooling to extract 2 × 2 (i.e. the kernel size) feature maps of the maximum activations of each filter. The max-pooling layer only outputs the maximum value of its respective convolutional layer outputs. The function of this max-pooling process can be thought of as determining whether the motif modelled by the respective convolutional layer exists in the input sequence or not.

The *dropout layer* [10] is then used to randomly mask portions of its output to avoid overfitting. This is achieved by eliminating a random fraction of p (the probability that an element is dropped) hidden neurons while multiplying the remaining neurons by 1/p. For our implementation p was set to 0.5.

The final *output layer* consists of two neurons corresponding to the two classification results with softmax activation. The two neurons are fully connected to the previous layer. The deep learning CNN architecture was implemented using Tensorflow [27] and TF.learn [28].

### Model validation

The goal of the model validation is to assess the models thoroughly for prediction accuracy. In this study two evaluation strategies were adopted: 10-fold cross validation and independent test samples.

### 10- fold cross validation

10-fold cross validation is a model validation technique to assess how the results of a model will be generalized to an independent data set. In 10-fold cross validation, the data is first partitioned into 10 equal segments (or folds). Then, 10 iterations of training and validation are performed where in each iteration, 9 folds are used for training and a different fold of data is held out for validation. The benchmark dataset is used for this purpose.

### Independent test samples

An independent test sample is a set of data that is independent of the data used in training the model. In addition to the k-fold cross-validation, independent test samples with known BL were used to evaluate the classification model as well. Independent Datasets 1 and 2 (Additional files 1 and 2) were used for this purpose.

### Overfitting

One problem with using deep learning models is that they are prone to overfitting. Overfitting occurs when a model fits too well to the training data and is unable to generalize well. In this research, we incorporated several techniques to combat this problem. First, we used a simple convolutional neural network architecture with only one convolutional layer. This lowers the complexity of our model by minimizing the possible training parameters, giving our model fewer opportunities to overfit. Next, we employed sampling, feature selection, and embedding techniques to augment our data set. Then, we used L2 regularization and dropout with the probability 0.5. Also, our model was tuned using 10-fold cross validation during training to determine how well our model performed at predicting independent samples. Lastly, our method performs very well when evaluating our independent dataset; this further demonstrates that our model is not overfitting. Additional file 3: Figures S1-S6 show the validation loss curves for each classifier.

### Evaluation metrics

As discussed earlier, the BL classification is presented as a 2-level predictor. In the first level given a protein sequence, we predict whether that sequence is a BL or not and in the next level we predict to which class the BL belongs. The novelty of the approach is that we have implemented both the molecular classes and the functional groups. As both molecular class and functional groups contain more than two classes, CNN-BLPred uses the one-vs.-rest strategy to solve this multi-class classification problem. By doing so, the CNN-BLPred assigns for each class either positive or negative to the test sequence, giving rise to four frequencies: true positive (TP), false positive (FP), true negative (TN), and false negative (FN).

The above four frequencies are then used to calculate various evaluation metrics. The metrics include accuracy, sensitivity, specificity, and Matthew’s correlation coefficient (MCC) and are defined below.

4 $$ \mathrm{Accuracy}=\frac{\mathrm{TP}+\mathrm{TN}}{\mathrm{TP}+\mathrm{TN}+\mathrm{FP}+\mathrm{FN}}\kern0.5em \times 100 $$

5 $$ \mathrm{Sensitivity}=\frac{\mathrm{TP}}{\mathrm{TP}+\mathrm{FN}}\times 100 $$

6 $$ \mathrm{Specificity}=\frac{\mathrm{TN}}{\mathrm{TN}+\mathrm{FP}}\times 100 $$

7 $$ \mathrm{MCC}=\frac{\left(\mathrm{TP}\right)\left(\mathrm{TN}\right)-\left(\mathrm{FP}\right)\left(\mathrm{FN}\right)}{\surd \left(\mathrm{TP}+\mathrm{FP}\right)\left(\mathrm{TP}+\mathrm{FN}\right)\left(\mathrm{TN}+\mathrm{FP}\right)\left(\mathrm{TN}+\mathrm{FN}\right)} $$

The area under the ROC (Receiver Operating Characteristic) curve (AUC) is also used as one of the metrics. We also compared our CNN-BLPred method with the existing PredLactamase [11]. The results of cross-validation were adopted from the PredLactamase paper and the results for the independent datasets were obtained using their web-server [11].

## Results

### Feature importance and feature selection

**Fig. 4:**
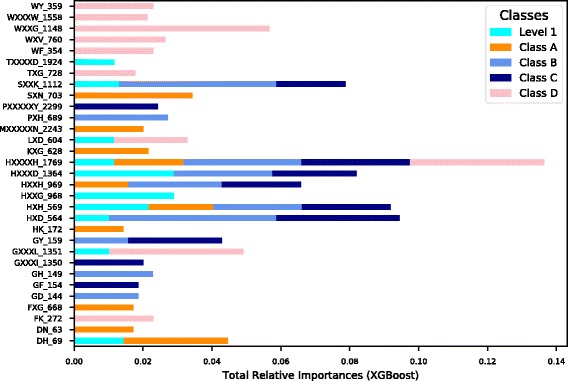
Top 10 Features from CKSAAP (*k-spaced Amino Acid Pairs*). The features and their relative importance after feature selection for Level 1 and Classes A, B, C and D using XGBOOST

As discussed in the methods section, feature importance is calculated based on a relative importance measure created by constructing gradient boosted trees. Any feature with a relative importance value of <0.001 is considered unimportant. Based upon this value, we were able to classify ~97.5% of the total features as unimportant and subsequently remove them. Figure 4 shows the top 10 features calculated from our classification models. Upon further analysis, we observed that features related to the Histidine (H) residue were heavily represented among the top features, which agrees with a previously published study [29]. This study reported a signalling system in which membrane-associated histidine kinase directly binds β-lactams, triggering the expression of a β-lactamase and resistance to β-lactam antibiotics. It is also interesting to note that features like WY, WXXXW, WXXG, WXV, WF and others were deemed important for Class D β-lactamase. This is in agreement with the observation that tryptophan plays a critical role for the activity and stability of class D β-lactamase [25].

### Performance of the individual feature type

**Fig. 5:**
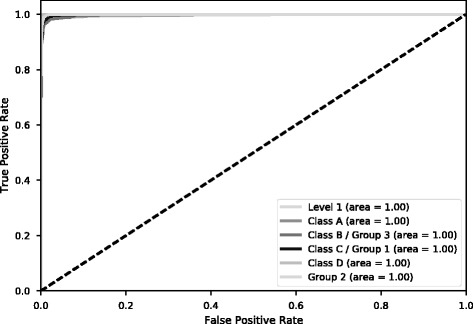
ROC Curve for 10-fold cross validation (CKSAAP). All curves follow closely to the left and top border, with AUC above 90%, indicating the classifiers have a high accuracy

**Fig. 6:**
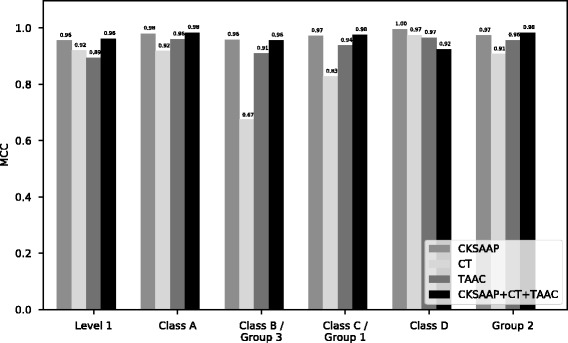
Comparison of MCC Scores based on 10-fold cross validation

In order to find the best combination of feature types, we compared the performance of individual features (i.e. CKSAAP, CT and TAAC) with the performance of the combination of all features (CKSAAP + CT + TAAC), which is represented in Table 5 as ALL. The performance of 10-fold cross validation for each of the features is shown in Table 5 for Level 1 prediction. A similar trend was observed for other class predictions. The performance of 10-fold cross validation and Independent Datasets 1 and 2 (Additional files 1 and 2) for other classes is shown in Additional file 3: Tables S4a-e. It can be observed from Table 5 that CKSAAP and the collective set have the best performance for 10-fold cross validation. CKSAAP also outperformed all other features for the independent test as indicated in Additional file 3: Tables S4a-e. The ROC curves for each of the features are shown in Fig. 5. From this evaluation, we determined that CKSAAP is the best feature set. Hence, only CKSAAP is used as the feature set for CNN-BLPred. The comparison of MCC scores for the 10-fold cross validation are presented in Fig. 6. We also show the performance of CKSAAP using 10-fold cross validation in Table 6. The performance of the independent test set of CKSAAP is shown in Table 7. In addition, other evaluation metrics like Sensitivity, Specificity, Accuracy, F1 score, MCC and AUC of the CKSAAP are shown in Table 8. The complete results of CNN-BLPred training are shown in Additional file 3: Table S7.

**Table 5:** Performance of CKSAAP, TAAC, CT and ALL for Level 1 using 10-Fold CV (ALL refers to CKSAAP + CT + TAAC)

Methods	Level 1			
	AUC	Sen (%)	Sp (%)	MCC
CKSAAP	1.00	99.90	95.73	0.96
CT	0.98	98.30	93.81	0.92
TAAC	0.98	97.27	92.29	0.89
ALL	1.00	99.77	96.47	0.96

**Table 6:** Performance of CKSAAP using 10-Fold Cross Validation

Class/Group	AUC	Sen (%)	Sp (%)	MCC
Level 1	1.00	99.90	95.73	0.96
Class A	1.00	98.03	100.00	0.98
Class B/Group 3	1.00	97.94	97.94	0.96
Class C/Group 1	1.00	98.02	99.15	0.97
Class D	1.00	99.58	99.97	1.00
Group 2	1.00	97.44	99.93	0.97

**Table 7:** Independent Test Set Performance of CKSAAP

Class/Group	AUC	Sen (%)	Sp (%)	MCC
Level 1	0.96	97.60	68.18	0.70
Class A	0.99	76.92	98.68	0.78
Class B/Group 3	1.00	100.00	98.48	0.99
Class C/Group 1	0.99	86.49	99.21	0.89
Class D	1.00	83.33	100.00	0.91
Group 2	0.99	89.47	96.55	0.81

**Table 8:** Complete Results of CNN-BLPred Independent Testing

Class	Sensitivity	Specificity	Accuracy	F1 Score	MCC
Level 1	97.60	68.18	94.18	0.97	0.70
Class A	76.92	98.68	96.95	0.80	0.78
Class B/Group 3	100.00	98.48	99.39	0.99	0.99
Class C/Group 1	86.49	99.21	96.34	0.91	0.89
Class D	83.33	100.00	99.39	0.91	0.91
Group 2	77.27	98.59	95.73	0.83	0.81

### Performance of the CNN-BLPred

We compared CNN (using CKSAAP as the feature based on the results in previous section) to other popular machine learning algorithms. Essentially, we compared the performance of CNN using our simple architecture with other machine learning methods like Random Forest and other Deep Learning architectures like RNN (Recurrent Neural Networks). In addition, we changed the architecture of our original Convolutional Neural Network (CNN) by adding another convolutional layer and max pooling layer after the original max pooling layer. We call this approach CNN-Ext. We also compared CNN-BLPred with PredLactamase. For the comparison of machine learning algorithms, we show results of both 10-fold cross validation as well as the independent test results (using Additional file 1: Independent Dataset 1) in Table 9. It was observed that CNN performed slightly higher than RF and significantly outperformed RNN and to some extent CNN-Ext. It must be noted that although CNN-Ext performs better in training (likely due to overfitting), it does not perform similarly in the independent set. In essence, with the comparison to other various ML algorithms and architecture, the one we used which is a simple architecture (with only one convolutional layer and max pooling layer) performs the best which supports the superior performance of CNN.

**Table 9:** Comparative Results using Benchmark Dataset 1 for RF, RNN, CNN-ext. and CNN. RF refers to Random Forest. RNN refers to Recurrent Neural Network. CNN-ext. refers to extended CNN where we use our original architecture with another convolutional layer and max pooling layer adding after the original max pooling layer. CNN refers to the Convolutional Neural Network described in the paper

#	Class/Group	Training				Independent Test			
		RF	RNN	CNN-ext	CNN	RF	RNN	CNN-ext	CNN
1	Level 1	0.97	0.43	0.95	0.96	0.95	0.70	0.69	0.70
2	Class A	0.97	0.16	0.97	0.98	0.75	0.70	0.78	0.78
3	Class B/Group 3	0.94	−0.04	0.96	0.96	0.94	0.34	1.00	0.99
4	Class C/Group 1	0.92	0.20	0.96	0.97	0.90	0.54	0.89	0.89
5	Class D	1.00	0.66	0.99	1.00	0.44	0.06	1.00	0.91
6	Group 2	0.96	0.42	0.97	0.97	0.75	0.34	0.81	0.81

**Fig. 7:**
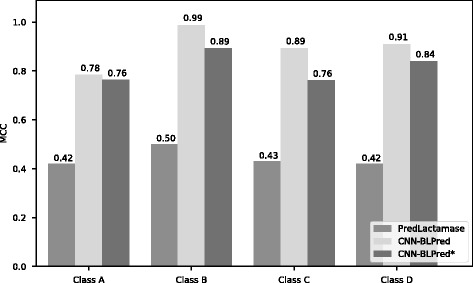
Comparison of PredLactamase vs. CNN-BLPred (Independent Test) using MCC on an independent test set. MCC score was higher using CNN-BLPred than PredLactamase. CNN-BLPred is testing using Independent Dataset 1 (Additional file 1). CNN-BLPred* is testing using Independent Dataset 2 (Additional file 2)

We only present the results of the independent test (the results of 10-fold cross validation showed similar trends). It was observed that for each class, a predictive MCC of at least 0.78 and overall MCC were obtained (overall MCC of 0.81 obtained for CNN-BLPred* and 0.89 obtained for CNN-BLPred). Interestingly, our prediction accuracy and MCC for non-BL was 94.18% and 0.70 respectively for CNN-BLPred. Fig. 7 shows the comparison between PredLactamase and our CNN-BLPred based on MCC scores.

#### Comparing PredLactamase with CNN-BLPred

The results of the independent test samples for our method CNN-BLPred and PredLactamase using Additional file 2: Independent Dataset 2 are summarized in Table 10, in the form of a confusion matrix. The column labelled ‘correct’ for both predictors show the number of sequences that were correctly identified while the one that is labelled ‘incorrect’ shows the number of sequences that were incorrectly predicted and the incorrectly predicted subfamily. The column ACC denotes the accuracy of each method in percentages. It was observed can be seen that for all the BL Classes and non-BL proteins, the accuracy of CNN-BLPred was higher than PredLactamase method for all the BL classes and non-BL proteins.

**Table 10:** Comparative Results using Independent Dataset 1 for PredLactamase and CNN-BLPred

Class	PredLactamase			CNN-BLPred*		
	Correct	Incorrect	ACC	Correct	Incorrect	ACC
A	15	5	75.00	18	2	90.00
B	15	5	75.00	19	1	95.00
C	15	5	75.00	18	2	90.00
D	15	5	75.00	19	1	95.00
Overall			75.00			92.50

*CNN-BLPred is testing using Independent Dataset 2

## Conclusions

We developed a Deep Learning based method (CNN-BLPred) to identify BL and subsequently classify them into respective BL classes. For the first time, in addition to molecular classes, we also implemented the functional classification. The BL classification problem is posed as a multi-class classification problem and solved using the one-vs.-rest strategy.

The number of embeddings were set to 128 based on the improved prediction accuracy. CNN-BLPred was able to predict with near optimal accuracy whether a query protein sequence belongs to one of the four molecular classes and/or one of the three functional groups. In order to use embedding technique effectively, this method uses CKSAAP features. This feature set was chosen, in part, because it can be represented as a small, continuous vector space and also because it outperformed other features that fit the same criteria (i.e. CT and TAAC).

To combat the class imbalance problem we used techniques such as ROS, RUS, and SMOTE. The number of features is considerably high compared to the number of sequences, which makes our classifier subject to the ‘curse of dimensionality’. To solve this issue we employ a feature selection method known as gradient boosted features selection.

Another concern we address is overfitting. Most overfitting problems are due to the fact that the dataset used for testing is used for the training as well. The training datasets used in this study were filtered from the closely similar and redundant sequences as explained in the dataset section. The test sequences, which were used for evaluation, are the sequences that were not included in the training dataset. A dataset with a redundancy reduction cut-off of 40% was utilized to ensure that our high prediction performance was not due to the sequence similarity of the dataset.

The method was systematically validated with cross validation and independent test samples using two sets of datasets that have varying sequence redundancy reduction criteria. Moreover, CNN-BLPred was compared with other machine learning algorithms like Random Forest, Recurrent Neural Network and other architectures of CNN and it was observed that a simple architecture of CNN works well for our purpose. Performance on the independent datasets and the comparative study between the CNN-BLPred and PredLactamase demonstrated that CNN-BLPred outperforms other well-established predictors. Deep Learning algorithms are considered to be better at learning abstract features from simple features, and one of the advantages of using Deep Learning is to get rid of hand-crafted features. The overall better performance of k-spaced amino acid features (a simple type of feature) also validates this point for this problem. Additionally, BL is a multi-domain protein and being able to identify a protein sequence as a BL will also help in prediction of its structure.

In conclusion, we were able to develop an improved BL classification method compared to existing methods based on Convolution Neural Network. A web site implementing the methodology will be developed soon to serve the scientific community. In the meantime, the software for the work is available upon request to academic researchers from the authors.

## Additional files

Additional file 1: Independent Dataset 1 (ZIP 5801 kb)

Additional file 2: Independent Dataset 2 (ZIP 604 kb)

Additional file 3: Figure S1. Validation/Loss curve for Level 1. **Figure S2.** Validation/Loss curve for Class A. **Figure S3.** Validation/Loss curve for Class B (Group 3). **Figure S4.** Validation/Loss curve for Class C (Group 1). **Figure S5.** Validation/Loss curve for Class D. **Figure S6.** Validation/Loss curve for Group 2. **Figure S7.** FEPS top 10 features for level 1 and Classes A, B, C, and D. **Table S1.** Molecular Class/Functional Group training dataset. **Table S2.** Molecular Class/Functional Group Independent Dataset 1. **Table S3.** Molecular Class/Functional Group Independent Dataset 2. **Table S4.** a-f Performance of CKSAAP, TAAC, CT and ALL for level 1 and classes A, B, C, and D using 10-fold CV. **Table S5.** a-f. Independent test set performance of CKSAAP, TAAC, CT and ALL for level 1 and classes A, B, C, and D. **Table S6.** a-d. Performance of CNN-BLPred with PredLactamase using independent test sets. **Table S7.** Complete results of CNN-BLPred training. (PDF 201 kb)

## Abbreviations

BL: Beta Lactamase
CNN: Convolutional Neural Network
NN: Neural network
RF: Random Forest
RNN: Recurrent Neural Network
ROS: Random over sampling
RUS: Random under sampling
SMOTE: Synthetic Minority Oversampling Technique
