# Supplementary material for: CNN-BLPred: a Convolutional neural network based predictor for β-Lactamases (BL) and their classes
Source: BMC Bioinformatics. 2017 Dec 28;18(Suppl 16):577. doi: 10.1186/s12859-017-1972-6 (PMC5751796; doi:10.1186/s12859-017-1972-6)
Supplement: Supplementary file 3 — Validation/Loss curve for Level 1. Figure S2. Validation/Loss curve for Class A. Figure S3. Validation/Loss curve for Class B (Group 3). Figure S4. Validation/Loss curve for Class C (Group 1). Figure S5. Validation/Loss curve for Class D. Figure S6. Validation/Loss curve for Group 2. Figure S7. FEPS top 10 features for level 1 and Classes A, B, C, and D. Table S1. Molecular Class/Functional Group training dataset. Table S2. Molecular Class/Functional Group Independent Dataset 1. Table S3. Molecular Class/Functional Group Independent Dataset 2. Table S4. a-f Performance of CKSAAP, TAAC, CT and ALL for level 1 and classes A, B, C, and D using 10-fold CV. Table S5. a-f. Independent test set performance of CKSAAP, TAAC, CT and ALL for level 1 and classes A, B, C, and D. Table S6. a-d. Performance of CNN-BLPred with PredLactamase using independent test sets. Table S7. Complete results of CNN-BLPred training. (PDF 201 kb) [file 12859_2017_1972_MOESM3_ESM.pdf]

Additional File 3

Supplementary Information Tables:

Supplementary Table S1. Molecular Class/Functional Group Training Dataset.

#	Class/Group	# of Sequences
1	Class A	268
2	Class B/Group 3	2069
3	Class C/Group 1	701
4	Class D	59
5	Group 2	318
6	Non BL	478

Supplementary Table S2. Molecular Class/Functional Group Independent Dataset 1.

#	Class/Group	# of Sequences
1	Class A	10
2	Class B/Group 3	115
3	Class C/Group 1	43
4	Class D	3
5	Group 2	22
6	Non BL	19

Supplementary Table S3. Molecular Class/Functional Group Independent Dataset 2.

#	Class	# of Sequences
1	Class A	4
2	Class B/Group 3	6
3	Class C/Group 1	6
4	Class D	4
5	Group 2	8

Supplementary Table S4a. Performance of CKSAAP, TAAC, CT and ALL for Level 1 using 10-Fold CV (ALL refers to CKSAAP+CT+TAAC).

Methods	Level 1			
	AUC	Sen (%)	Sp (%)	MCC
**CKSAAP**	**1.00**	**99.90**	**95.73**	**0.96**
CT	0.98	98.30	93.81	0.92
TAAC	0.98	97.27	92.29	0.89
ALL	1.00	99.77	96.47	0.96

Supplementary Table S4b. Performance of CKSAAP, TAAC, CT and ALL for Class A using 10-Fold CV.

Methods	Class A			
	AUC	Sen (%)	Sp (%)	MCC
**CKSAAP**	**1.00**	**98.03**	**100.00**	**0.98**
CT	0.99	92.56	99.50	0.92
TAAC	1.00	96.68	99.38	0.96
ALL	1.00	98.40	99.96	0.98

Supplementary Table S4c. Performance of CKSAAP, TAAC, CT and ALL for Class B/Group 3 using 10-Fold CV.

Methods	Class B/Group 3			
	AUC	Sen (%)	Sp (%)	MCC
**CKSAAP**	**1.00**	**97.94**	**97.94**	**0.96**
CT	0.91	84.87	82.62	0.67
TAAC	0.99	95.58	95.40	0.91
ALL	1.00	97.93	97.65	0.96

Supplementary Table S4d. Performance of CKSAAP, TAAC, CT and ALL for Class C/Group 1 using 10-Fold CV.

**Methods**	**Class C/Group 1**			
	**AUC**	**Sen (%)**	**Sp (%)**	**MCC**
**CKSAAP**	**1.00**	**98.02**	**99.15**	**0.97**
CT	0.97	89.16	93.84	0.83
TAAC	1.00	96.48	97.30	0.94
ALL	1.00	98.06	99.49	0.98

Supplementary Table S4e. Performance of CKSAAP, TAAC, CT and ALL for Class D using 10-Fold CV.

**Methods**	**Class D**			
	**AUC**	**Sen (%)**	**Sp (%)**	**MCC**
**CKSAAP**	**1.00**	**99.58**	**99.97**	**1.00**
CT	1.00	97.78	99.70	0.97
TAAC	1.00	97.57	99.00	0.97
ALL	1.00	96.04	96.38	0.92

Supplementary Table S4f. Performance of CKSAAP, TAAC, CT and ALL for Group 2 using 10-Fold CV.

**Methods**	**Group 2**			
	**AUC**	**Sen (%)**	**Sp (%)**	**MCC**
**CKSAAP**	**1.00**	**97.44**	**99.93**	**0.97**
CT	0.98	91.94	99.07	0.91
TAAC	1.00	96.48	99.22	0.96
ALL	1.00	98.31	100.00	0.98

Supplementary Table S5a. Independent Test Set Performance of CKSAAP, CT, TAAC and ALL for Level 1.

**Methods**	**Level 1**			
	**AUC**	**Sen (%)**	**Sp (%)**	**MCC**
**CKSAAP**	**0.96**	**97.60**	**68.18**	**0.70**
CT	0.84	95.09	42.31	0.43
TAAC	0.91	97.44	45.45	0.54
ALL	0.96	98.80	77.27	0.81

Supplementary Table S5b. Independent Test Set Performance of CKSAAP, CT, TAAC and ALL for Class A.

**Methods**	**Class A**			
	**AUC**	**Sen (%)**	**Sp (%)**	**MCC**
**CKSAAP**	**0.99**	**76.92**	**98.68**	**0.78**
CT	0.71	20.00	93.96	0.15
TAAC	0.97	75.00	98.03	0.73
ALL	0.98	76.92	98.68	0.78

Supplementary Table S5c. Independent Test Set Performance of CKSAAP, CT, TAAC and ALL for Class B/Group 3.

**Methods**	**Class B/ Group 3**			
	**AUC**	**Sen (%)**	**Sp (%)**	**MCC**
**CKSAAP**	**1.00**	**100.00**	**98.48**	**0.99**
CT	0.92	85.05	85.96	0.69
TAAC	0.99	95.96	93.85	0.90
ALL	1.00	98.99	98.46	0.97

Supplementary Table S5d. Independent Test Set Performance of CKSAAP, CT, TAAC and ALL for Class C/Group 3.

**Methods**	**Class C/Group 1**			
	**AUC**	**Sen (%)**	**Sp (%)**	**MCC**
**CKSAAP**	**0.99**	**86.49**	**99.21**	**0.89**
CT	0.92	59.18	96.52	0.64
TAAC	0.98	83.78	98.43	0.86
ALL	0.99	88.24	97.69	0.87

Supplementary Table S5e. Independent Test Set Performance of CKSAAP, CT, TAAC and ALL for Class D.

**Methods**	**Class D**			
	**AUC**	**Sen (%)**	**Sp (%)**	**MCC**
**CKSAAP**	**1.00**	**83.33**	**100.00**	**0.91**
CT	0.92	40.00	98.11	0.38
TAAC	0.97	44.44	99.35	0.58
ALL	1.00	100.00	99.38	0.89

Supplementary Table S5f. Independent Test Set Performance of CKSAAP, CT, TAAC and ALL for Group 2.

**Methods**	**Group 2**			
	**AUC**	**Sen (%)**	**Sp (%)**	**MCC**
**CKSAAP**	**0.99**	**89.47**	**96.55**	**0.81**
CT	0.90	60.87	94.33	0.56
TAAC	0.98	76.00	97.84	0.78
**ALL**	**1.00**	**100.00**	**97.93**	**0.92**

Supplementary Table S6a. Class A Performance Comparison of CNN-BLPred with PredLactamase using Independent Test Sets. CNN-BLPred refers to the results on our independent test set (Independent Dataset 1). CNN-BLPred* refers to PredLactamase's independent test set (Independent Test Set 2).

**Methods**	**Class A**		
	**Sen (%)**	**Sp (%)**	**MCC**
PredLactamase	75.00	75.00	0.42
CNN-BLPred	76.92	98.68	0.78
CNN-BLPred*	66.67	100.00	0.76

Supplementary Table S6b. Class B/Group 3 Performance Comparison of CNN-BLPred with PredLactamase using Independent Test Sets.

**Methods**	**Class B/Group 3**		
	**Sen (%)**	**Sp (%)**	**MCC**
PredLactamase	83.33	71.43	0.50
CNN-BLPred	100.00	98.48	0.99
CNN-BLPred*	85.71	100.00	0.89

Supplementary Table S6c. Class C/Group 1 Performance Comparison of CNN-BLPred with PredLactamase using Independent Test Sets.

**Methods**	**Class C/Group 1**		
	**Sen (%)**	**Sp (%)**	**MCC**
PredLactamase	66.67	78.57	0.43
CNN-BLPred	86.49	99.21	0.89
CNN-BLPred*	83.33	92.86	0.76

Supplementary Table S6d. Class D Performance Comparison of CNN-BLPred with PredLactamase using Independent Test Sets.

Methods	Class D		
	Sen (%)	Sp (%)	MCC
PredLactamase	75.00	75.00	0.42
CNN-BLPred	83.33	100.00	0.91
CNN-BLPred*	100.00	94.12	0.84

Supplementary Table S7. Complete Results of CNN-BLPred Training.

Class	Sensitivity	Specificity	Accuracy	F1 Score	MCC	AUC
Level 1	99.90	95.73	97.72	0.98	0.96	1.00
Class A	98.03	100.00	99.00	0.99	0.98	1.00
Class B/Group 3	97.94	97.94	97.94	0.98	0.96	1.00
Class C/Group 1	98.02	99.15	98.58	0.99	0.97	1.00
Class D	99.58	99.97	99.77	1.00	1.00	1.00
Group 2	99.93	97.38	98.65	0.99	0.97	1.00

Supplementary Figures:

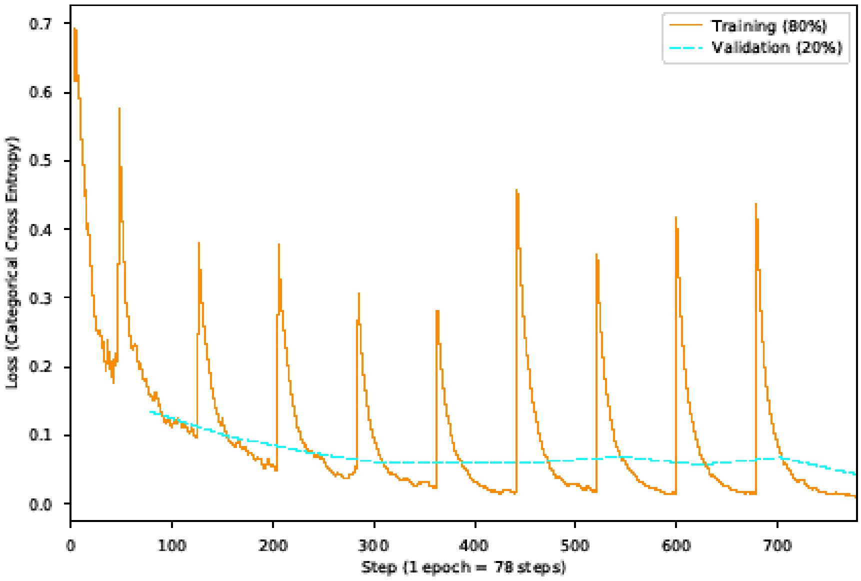

Supplementary Figure S1: Level 1 - Validation/Loss Curve

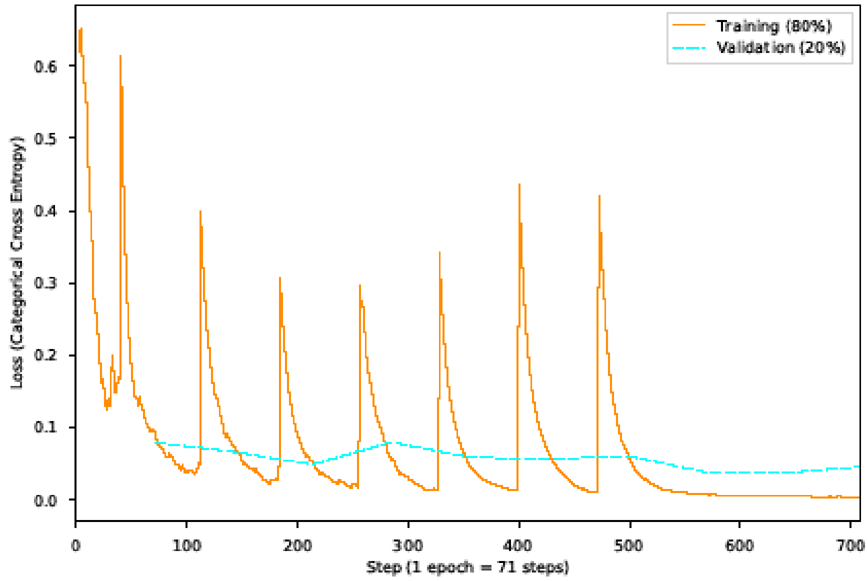

Supplementary Figure S2: Class A – Validation/Loss Curve

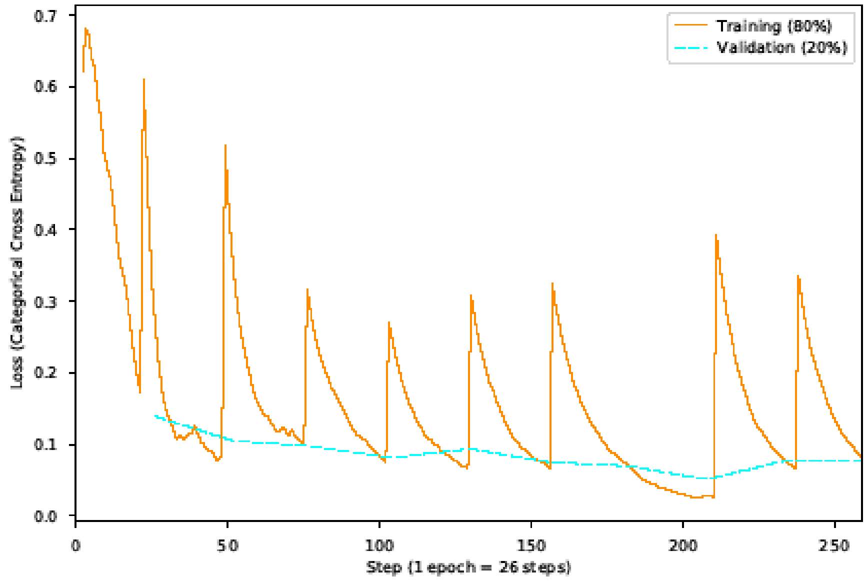

Supplementary Figure S3: Class B (Group 3) – Validation/Loss Curve

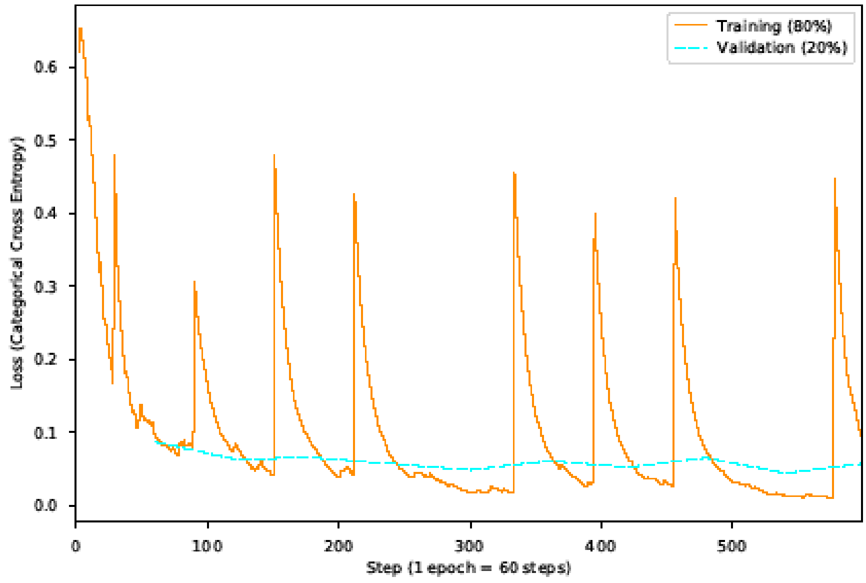

Supplementary Figure S4: Class C (Group 1) – Validation/Loss Curve

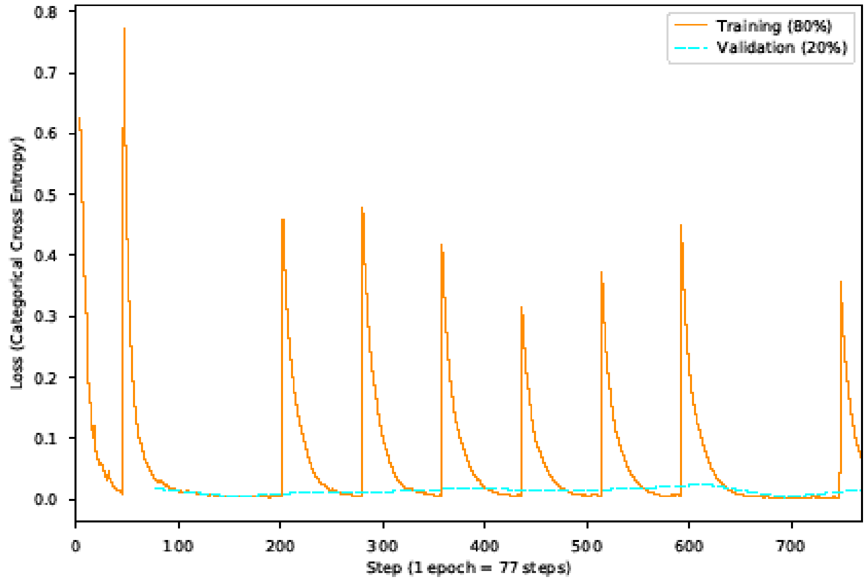

Supplementary Figure S5: Class D – Validation/Loss Curve

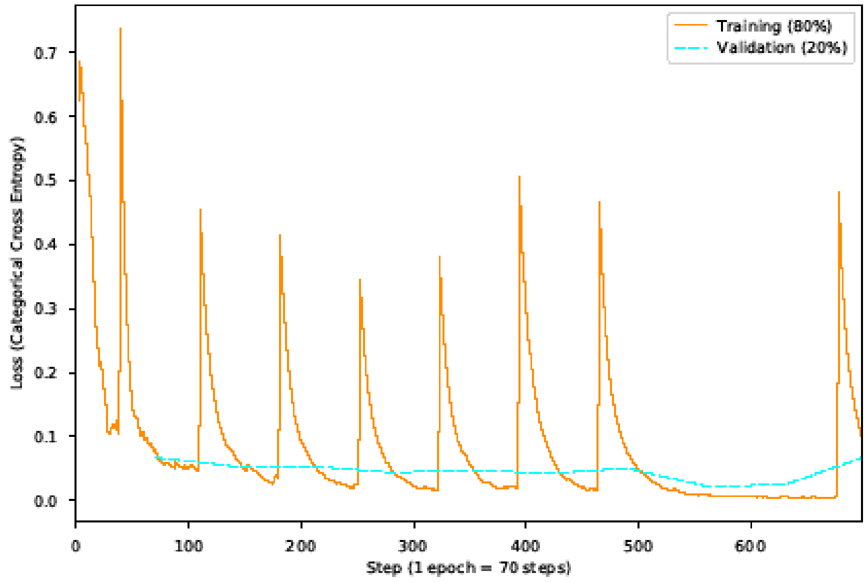

Supplementary Figure S6: Group 2: Validation/Loss Curve

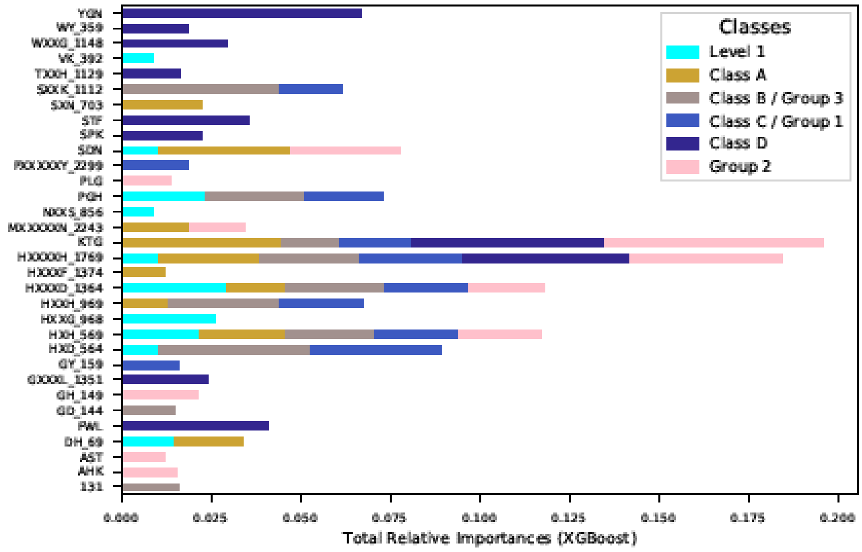

Supplementary Figure S7: FEPS top 10 Features for Level 1 and Classes A, B, C and D. WY, WXXG, VK, TXXH, SXXK, SXN, PXXXXXY, NXXS, MXXXXXN, HXXXXH, HXXXF, HXXXD, HXXH, HXXG, HXH, HXD, GY, GXXXL, GD and DH are CKSAAP features. YGN, STF, SPK, SDN, PGH, KTG and FWL are TAAC features. 131 are CT features.
